# Insights into parents’ perceived worry before and during the COVID-19 pandemic in Australia: inequality and heterogeneity of influences

**DOI:** 10.1186/s12889-023-16337-9

**Published:** 2023-10-07

**Authors:** Roula Zougheibe, Ashraf Dewan, Richard Norman, Ori Gudes

**Affiliations:** 1 School of Earth and Planetary Sciences, Curtin University, Kent Street, Perth, WA 6102 Australia; 2 School of Population Health, Curtin University, Perth, WA Australia; 3 School of Population Health, UNSW Medicine, New South Wales, Australia

**Keywords:** Pre-and-during COVID-19, Parents’ worry, Inequality, Regression analyses, Australia, Conceptual framework, GIS

## Abstract

**Background:**

Excessive worry is an invisible disruptive force that has adverse health outcomes and may advance to other forms of disorder, such as anxiety or depression. Addressing worry and its influences is challenging yet crucial for informing public health policy.

**Methods:**

We examined parents’ worries, influences, and variability before and during COVID-19 pandemic and across geography. Parents (n = 340) and their primary school-aged children from five Australian states completed an anonymous online survey in mid-2020. After literature review, we conceptualised the influences and performed a series of regression analyses.

**Results:**

Worry levels and the variables contributing to parents’ worry varied before to during the pandemic. The proportion of parents who were "very worried all the time" increased by 14.6% in the early days of the pandemic. During the pandemic, ethnic background modified parents’ worry and parents’ history of daily distress symptoms was a significant contributor (p < 0.05). Excessive exposure to news remained significant both before and during the pandemic. The primary predictor of parents’ worry before COVID-19 was perceived neighbourhood safety, while the main predictor during COVID-19 was financial risk due to income change. Some variable such as neighbourhood safety and financial risk varied in their contribution to worry across geographical regions. The proportion of worried children was higher among distraught parents.

**Conclusion:**

Parents’ worry during the health pandemic was not triggered by the health risks factors but by the financial risk due to income change. The study depicts inequality in the impact of COVID-19 by ethnic background. Different policies and reported virus case numbers across states may have modified the behaviour of variables contributing to the geography of parents’ worry. Exposure to stressors before the COVID-19 pandemic may have helped parents develop coping strategies during stressful events. Parents are encouraged to limit their exposure to stressful news. We advocate for parents-specific tailored policies and emphasise the need for access to appropriate mental health resources for those in need. Advancing research in geographical modelling for mental health may aid in devising much-needed location-targeted interventions and prioritising resources in future events.

**Supplementary Information:**

The online version contains supplementary material available at 10.1186/s12889-023-16337-9.

## Introduction

The World Health Organisation (WHO) defines human well-being as the integration of physical, social and mental health [1]. Epidemic diseases pose a constant threat to global health [2]. However, beyond physical health, public health crises have also been reported to cause various forms of social, emotional, and mental distress [3] which can be particularly heightened among vulnerable populations. Constant thinking, when danger or threats arises, can escalate into excessive worry, an invisible force that may disrupt human health. There is ample evidence in the literature indicating that increased worry can progress to anxiety [4] and potentially develop into disorders with stress-related symptoms or depression [5]. This can have consequences on parents who are exposed to an array of stressors that have originating outside their system [6]. Uncertainty about the future may exacerbate worry within families [7]. Yet, the impact is not uniformly distributed, resulting in varying degrees of anticipating an event as stressful.

When coronavirus (SARS-COV-2; COVID-19) landed in Australia, families were recovering from unprecedented bushfires [8–10] that caused substantial environmental damage [11] and loss of property. Highened worry, stemming from concerns about health safety and the future, may have already been prevalent among the population that had experienced this natural disaster. On the other hand, throughout the pandemic, compared to other countries, Australia made up a small proportion of both global cases and fatality rates. From the onset of the pandemic till the time of writing this manuscript, 99.1% of Australians who contracted the virus reported mild to moderate symptoms, with only 0.17% of the confirmed cases being severely ill [12]. Yet, uncertainty about the future has likely generated feelings that could negatively affect people’s thoughts, emotions and behaviour [13]. Additionally, disruption of life behaviour in terms of social distancing and lockdowns to curb virus transmission and economic hardships may have adversely threatened population well-being, with commonalities across the world [14, 15].

Drawing attention to the impact of health crises beyond physical health is challenging. Yet, research needs to systematically unpack key dimensions of settings most relevant for mitigating these impacts and guiding appropriate policies. Worry addressed in past studies on health crises like H1N1 and Ebola [16] or in mass violence in the US [17] have found that such events lead to excessive population worry, that can significantly impact the quality of people’s life [18]. The prevalence of anxiety and distress disorder [19] is often associated with increased worry. While some scholars have addressed the increase in vulnerability [20] and worry [14, 21] during the pandemic, their focus has primarily been on the adult population.

There is growing body of research that pays attention to the links between places and adverse effects on human health [22]. Spatial intelligence can help unfold research questions such as “where” and “why”. Informing public health policy when modelling spatial relationships and explaining factors behind observed spatial patterns [23] is feasible. Addressing the relation of place to population worry has been explored in past studies on environmental hazard [7, 24]. The recent pandemic saw an unprecedented use of applications such as dashboards to map the worldwide spread of COVID-19 cases [25–27] or adopt of case tracing using digital solutions to support a safe society in many countries [28]. However, lacking knowledge on the deleterious consequences of the recent COVID-19 or past pandemics beyond physical health, especially among specific population group of parents [19] and the geographical dimensions, has prompted scholars to call for further work in these settings.

Examining the relationship between parents’ worry and potential influences may aid in recognising the characteristics of vulnerable parents during abnormal events [7] and provide tools to mitigate the impact of pervasive pathological worry. Unlike past studies on people’s worry that employ a global static model of the variables examined [19, 29], addressing the spatial behaviour of variables in their local form may help avoid potential biases in the outcome and policy implications. No research has yet explored variations of influences on parents’ worry in everyday life compared to those triggered by an abnormal event such as health crises. Past studies indicate that parental levels of worry may vary depending on the age of the children they care for [30]. Therefore, addressing parents’ worry while focusing on one specific age groups of children is likely to help prevent over-generalisation in the outcomes.

### Conceptualising parents worry during or before health crises

**Fig. 1:**
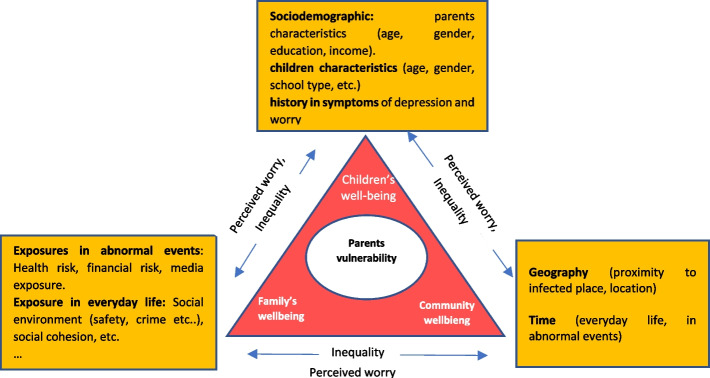
Overview of the conceptual framework of parents ‘ worry in a health crisis adapted from Chen, 2021 “risk triangle”

After a review of existing literature at the intersection of health geography, worry and health crises, we adapt the study by Prior et al. (2019) to address the general form of worry. Additionally, we utilised Crichton’s "risk triangle" technique, originally employed to assess perceived risk in natural disasters [31]. This triangle framework encompasses key components of natural hazards, vulnerability, and elements at risk, each placed at one of its corner. Over time, the technique evolved to incorporate spatial associations while examining factors related to community resilience [32]. In our study, we grouped factors contributing to parents’ vulnerability to worry and placed each group on one side of the triangle, Fig. 1. The fear of contracting the virus during the pandemic has intensified, as has the uncertainty regarding financial income [33], both of which we address in this study. Additionally, the relationship between worry and other variables perpetuates over the time and across geography dimension, placed at one of the corners of the triangle. Proximity to the source of threat, for instance, may disproportionately contribute to parents’ perceived risk or worry. Furthermore, the level of impact is dynamic and likely to differ between regular times and abnormal events such as health crises. For example, perceived neighbourhood safety may trigger worry in everyday life while other factors, such as media exposure, may play a more prominent role during abnormal events [17, 34]. Detailed attributes of individual characteristics, socioeconomic status, a history of distress, and other variables deemed important from past literature are discussed in the following section.

Additionally, several dimensions are likely to lead to varying the impact of these variables. The red area inside the triangle, Fig. 1, illustrate the implications on population well-being, extending to families, children and communities. The circle within the triangle represents parents’ vulnerability to worry. A larger circle denotes a higher vulnerability that is likely to elevate risks to family, children, and the community well-being (depicted in red within the triangle). Research has warned about the effect of increased parental worry on their own well-being [35], their ability to look after their children [36], heightened concerns straining the parent–child relationship [6], and the risk of child abuse [35].

To this end, this paper aims to fill a void in research by advancing our understanding of the characteristics of parents -who care for primary school-aged children (grade 4–6)- who experience heightened levels of worry. We explore stressors that contribute to this heightened worry, both within and outside the context of a disease outbreak. We signify the heterogeneity of exposures over time (i.e. variation of factors contributing to parents’ worry between everyday life and during abnormal events) and across geographical locations. The outcome of this study can aid in the early detection and mitigation of the adverse impact of worry on family structures and enhance preparedness in terms of public health resources for future events. In thist regard, our research seeks to address the following questions:

- (RQ1) What factors exacerbate parents’ worry in both everyday life and during health crises such as COVID-19?
- (RQ2) How does geography modify the factors influencing parental worry before and during the pandemic?
- (RQ3) In everyday life, what variables are most important in predicting heightened parental worry, and how does these predictors change during the pandemic? Finally
- (RQ4) Do worried children reside with worried parents?

## Methods

### Study areas and settings

**Fig. 2:**
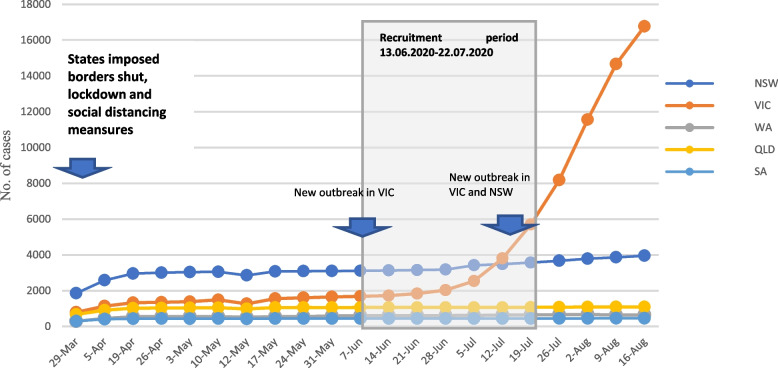
The COVID-19 situation in the five Australian states during recruitment regarding the number of cases, outbreaks, and imposed measures between 29th March 2020 and 16th August 2020

The study launched an anonymised online survey during the early stages of the pandemic (from June to July 2020) using Qualtrics^XM^ survey software [37]. We conducted surveys in five Australian states: New South Wales (NSW), Victoria (VIC), Queensland (QLD), South Australia (SA) and Western Australia (WA), which collectively represent 95% of Australian population. Notably, areas with a high number of coronavirus and mortality rates align with the high population density such as NSW and VIC during the pandemic [12]. Early measures, including border closure, were implemented to combat the outbreak. The state of VIC, for example, emerged on the 8^th^ of September 2021 from the most extended lockdown of 200 continuous days, with cases rising daily. At the time of the survey, WA had lifted its lockdown, but maintained social distancing measures. The eastern states of NSW and VIC were experiencing a second outbreak, Fig. 2.

### Ethics approval, consent and participants’ recruitment

Following university ethical approval (Ref: HRE2018-0683), we completed a soft survey launch to ensure the questionnaires’ functionality and clarity. Next, we recruited parents and children from WA who voluntarily participated in the survey through various channels, including social media (i.e., Facebook, Twitter, and university communications), email, and text messages. The invitation contained a link to the survey. For the remaining states, we collaborated with the GrowthOps research company, that offered both parents and their children (via their parents) a small cash incentive upon survey completion. Information regarding the research objectives and significance is loaded at the start of the survey. Unreliable, incomplete, or missing entries (at least one field) or completed in less than the estimated time of completing the survey (15–20 min average time is driven from entries during the pilot survey) were excluded. Internal IDs for accepted entries were communicated to the research company to release the incentives. To continue in the survey, parents or legal guardians must pass the screening questions of (1) residing in one of the five states, (2) having a child in the nominated age group, and consent to complete the survey to the best of their knowledge. The survey comprises three parts; the first is to complete by the parents, a parent and the child jointly fill the second part, and the child independently answers the last part under the parents’ supervision and assistance if needed.

## Measures

### Independent variables

#### Socioeconomic characteristics

Age, gender, education, income, and ethnic composition of individuals are found to magnify inequalities and anxiety during disease outbreaks [38–40]. Parents’ self-reported their sociodemographic characteristics included age (for analysis grouped into 25–34, 35–44, 45–54, and 55–64 years), gender (male, female, other), educational attainment (University graduate, undergraduate, high school or less, vocational/technical), and monthly household income before the pandemic (categories ranged from "$3,500 or less" to "over 6,500$"). We also asked parents about their country of birth and the first and second language spoken at home for ethnic background. Each child reported their grade (4, 5 and 6), gender (male, female, others), and school type (private or public). The "decline to answer" option was included in each question and was later re-coded as undisclosed.

#### Pre-COVID-19 stressors

Pre-existing anxiety and distress symptoms shape behavioural responses to abnormal events [16, 41]. Perception of safety in the neighbourhood modifies people’s behaviour in regular life and can promotes psychological distress [42]. Perceived neighbourhood safety in the week before the outbreak was reported by parents on a 5-point Likert scale (1 = Not sure, 2 = Not at all safe, 3 = Quite Unsafe, 4 = Quite Safe, 5 = Very Safe). We employed the SF-36 health survey form to identify people with a history of distress and worry symptoms. This tool proved reliable in assessing people’s mental or physical health, among other areas [43]. Parents were asked whether they experienced (a) feeling distressed or (b) being anxious or if their children experienced distress or disinterest in playing in the month before the pandemic. Each item is rated on a 4-point Likert scale (from Not at all to Nearly every day). Exposure time (in hours) to the news in the week before the pandemic outbreak and the frequency (in days) from (1 = Never, 2 = Less than 30 min a day, 3 = Less than an hour a day, 4 = 1–3 h a day, 5 = four or more hours a day) among parents is reported. Despite not being considered in the current study, there is evidence in the literature of the positive adverse effect of neighbourhood cohesion in pre-pandemic [44] and social support during a health crisis [21].

#### COVID-19-related stressors

The fragile pandemic-related settings of (a) changes in financial status and (b) fear of health risk from the virus were examined. Parents reported their exposure to COVID-19 health risk, categorised as: 1 = direct exposure to the virus, or members of the family was infected (high risk), 2 = knew someone infected in the building or the local area (moderate risk), and 3 = no direct exposure (low risk). We also assessed the actual change, due to the pandemic, in the primary household’s financial status (1 = No, it is about the same but work from home, 2 = No, it is about the same and goes to work in-person, 3 = Salary is reduced, 4 = Seeking employment, and 5 = Jobkeeper or if none of above they can select 5 = Other and explained further the changes). During a global crisis, information is crucial as people rely on it to understand their daily situations. At the same time, research suggests that high exposure to news can exacerbates anxiety and fear [16, 21, 45]. A 5-point Likert-type scale was used (ranging from "never" to "four or more hours a day") to capture parents’ and children’s time and frequency following the news during the pandemic [16, 17].

#### Response variable

We assessed parents’ perceived worry, using a 5-point Likert-type scale, ranging from "not at all worried" to "very worried all the time" [16], over two weeks: one week during the pandemic (we specified the first week of the outbreak) and one week prior to the COVID-19 outbreak. Children independently from their parents rated their COVID-19-related worry of becoming infected or playing out.

Spatial data: surveyed points data of this study were aggregated to the postal area address (POA) units.

### Analyses

**Fig. 3:**
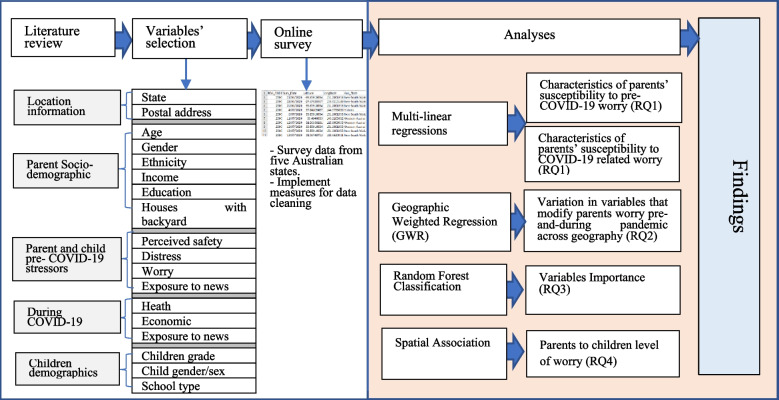
Analysis workflow for modeling parents' worry during and before the pandemic. RQ# denote number of research question addressed in this paper

The analytical method in Table S1, Additional File 1 and Fig. 3 illustrate the workflow of the analyses conducted. Collected data were assessed, cleaned, and converted into a point feature layer using the common POA field. This is a prerequisite step for carrying out the necessary spatial analyses. Following descriptive and bivariate analyses, we identify variables that modify parents’ worry (RQ1) using multilinear regressions, a global modelling approach. Variables’ levels were factored in, that conceptually categorises the data into levels [46]. We assigned a reference level for each variable [21]. To determine if assumptions of multiple linear regressions are met, we created a scatter plot matrix to assess each predictor variable and the response variable’s linear relationship. We used a Q-Q plot to check the normality of data distribution. We execute variance inflation factors (VIFs) to assess redundancy or overlapping among variables (Chen et al., 2021). We used the Akaike information criterion (AIC) score for model evaluation.

Global models assume no variation over space in the relationships between explanatory and dependent variables [6]. Yet they are useful as a base model to compare to other models [47]. Model1 during COVID-19 and Model2 in pre- COVID-19 were regressed. Both models have a general form:

1 $${y}_{i} = {\beta }_{0} +{ \beta }_{1x1} + \dots ..+ {\beta }_{nxn}+ \varepsilon$$

where y is the response variable of parents’ COVID-19 worry at country or state *i,* β_0_ denotes the intercept, β_1_ is the slope parameter, (X_1_, X_2_,.., X_n_) are independent explanatory variable(s), and ε is the error term. As bivariate and regression analyses depicted the association of ethnic background with parents’ worry during the pandemic, we fitted two multiple linear regression models that controlled for parents’ ethnic backgrounds.

Next, we move away from spatial stationarity and explore spatial dependency among variables (RQ2) using the local form of linear regression of geographically weighted regression (GWR) [48, 49]. The varying coefficients generated for each of the geographic units in the analysis [50] explain how the variable in that location has contributed to predicting the response variable. We used Poisson for discrete data with golden search option [51]. The GWR is denoted by [52].

2 $${Y}_{i}= {\beta }_{i0}+ \sum_{j=1}^{M}{\beta }_{ij}{X}_{ij}+{\varepsilon }_{i}, =1, 2, \cdots , N$$

where *y*_*i*_ is the dependent variable of parents’ worry in postal code area *i*; *β*_*i0*_ refers to regression intercept; *β*_*ij*_ is the value of the *j*th regression parameter, X_ij_ is the value of the *j*th explanatory parameter, and *εi* refers to regression error.

Variables’ importance (RQ3) in forest-based classification uses Gini coefficients [51] to compute the importance score for each selected variable to understand the degree of each variable in contribution to the random forest model [53]. Finally, for (RQ4), we used spatial association to depict the geographical correlation between worried parents and children [54]. Statistical analyses were conducted in RStudio 4.0.5, and spatial statistical analysis used ArcGIS Pro 2.8.3.

## Results

### Sample characteristics

A larger percentage of the 340 respondents were female parents (57%). Approximately 42% of the parents were under 35 years old. Using Australia’s Bureau of Statistics (ABS) broad ethnic grouping [55], 49% of participants were Oceanian by birth. The countries falling under this category are in listed in Table S2, Additional File 1. Parents’ education levels are 38.5% at or above bachelor “Graduate degree”, Undergraduates were 37.9%, high school or less were 15.2%, and 8.2% had vocational or technical qualifications. In terms of income, 22.9% of parents fell into the lowest income category of $3,499 monthly, 37.6% had median yearly income (between $3,400 to less than $6,500 monthly), and 32% had high income ($6,500 or more monthly). The study sample closely represented the general Australian population although the percentage of parents with undergraduate qualifications was slightly underrepresented compared to a figure of 48.6% reported in the ABS’s latest survey in 2018. Most parents reported no direct exposure to COVID-19 cases (78%). Nearly 56% reported retaining their jobs, whereas 27.4% experienced salary reduction due to the pandemic at the time of the survey. Additional descriptive statistics can be found in Table S3, Additional File 1.

### Bivariate relationships

**Fig. 4:**
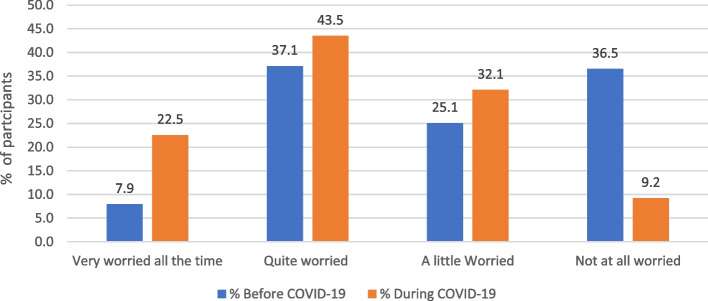
The percentage of parents reporting their level of worry: A comparison of before and during pandemic worry levels

Figure 4 shows a 14.6% increase in parents who reported “very worried all the time” and a decline of 27.3% in parents who reported “not worried at all” during COVID-19 compared to pre-pandemic.

Parents’ exposure to news soared during the pandemic, with 36% from 1–3 h of daily news consumption compared to 18% before the pandemic. The percentage of those reporting "more than four hours" of daily exposure has tripled, reaching 12% during the pandemic compared to 4% before the outbreak. Parents that never followed any news decreased from 7 to 1% in pre-and-during the pandemic comparison.

### Regressions results

#### Parents’ COVID-19-related worry

Model 1 in Table 1 presents a multi-regression model depicting a significant association of decreased parent worry when parents have Oceanian (p < 0.05) and European (p < 0.1) ethnic backgrounds compared to those categoirsed as “Asian and Middle-Eastern” (reference level). Increased parents’ worry is associated with lower perceived neighbourhood safety levels (p < 0.05) compared to those reported feeling very safe (reference level) and among parents with a history of daily distress symptoms. From COVID-19-related stressors, parents’ worry was positively associated with increased daily exposure to pandemic news "four hours and more daily" (p < 0.05). Income changes increased parental worry, particularly for those whose salary was reduced (p < 0.05).

**Table 1:** Hierarchical multilinear regressions of parents’ COVID-19 and pre-COVID-19 worry

Variable	During COVID-19	Before COVID
	**Model 1**	**Model 2**
***Sociodemographic:*** **Age (ref: **25-35)		
35–44	-0.045 (0.702)	0.430 (0.761)
45–54	-0.084 (0.139)	-0.218 (0.153)
55–65	-0.092 (0.178)	-0.215(0.196)
**Gender (ref: female)**		
Male	-0.000 (0.141)	**-0.260**^**#**^** (0.154)**
**Ethnicity (ref:** Asian, Middle Eastern)		
European	**-0.352**^**#**^** (0.182)**	0.154 (0.199)
Oceanian	-0.275* (0.117)	0.011 (0.127)
Others	**-0.477**^**#**^** (0.266)**	0.044 (0.284)
**Education (ref:** High school or less		
Vocational/technical	-0.108 (0.220)	0.009 (0.242)
Undergraduate	-0.016 (0.171)	0.083 (0.213)
Graduate	0.048 (0.175)	-0.039 (0.216)
**Household (ref:** $3,499 or less)		
$3,500—$6,499 (3)	0.125 (0.133)	0.0433 (0.144)
$6,500 or more (4)	-0.133 (0.145)	0.054 (0.156)
**Life stressors (History): Safety perception (ref:** Very Safe (1))		
Quite Safe	0.321* (0.126)	0.059 (0.254)
Quite Unsafe	-0.220 (0.152)	-0.055 (0.166)
Not at all safe	-0.145 (0.231)	0.052 (0.138)
**History of parents’ distress (ref:** Not at all)		
More than half the day	0.118 (0.221)	0.213 (0.238)
Several days	-0.041 (0.139)	0.073 (0.151)
Nearly every day	0.565* (0.258)	0.231 (0.278)
**History of parents’ worry (ref:** Not at all)		
More than half the day	-0.088 (0.238)	0.478* (0.242)
Several days	0.062 (0.151)	**0.301**^**#**^ **(0.158)**
Nearly every day	-0.266 (0.296)	0.459 (0.320)
**Parents’ Exposure to Media news before the pandemic** (ref: Less than 30 min a day 2)		
Never		-0.613** (0.224)
Less than an hour a day		-0.020 (0.130)
1–3 h a day		0.347* (0.162)
Four hours and more		0.347 (0.162)
**COVID-19-related life stressors: Income change (ref:** No, it is about the same, and normally go to work)		
No, it is about the same, but work from home mainly	-0.232 (0.335)	
Job-keeper	**-0.162 (0.219)**	
Salary is reduced	-0.799* (0.373)	
Seeking employment	0.415 (0.601)	
**Exposure to COVID-19 infection** (**ref**: No direct exposure)		
Direct exposure (being infected or someone in the family being infected)."	-0.043 (0.189)	
"Having a close relationship with someone infected in the buildings or local area."	-0.029 (0.198)	
**Parents’ exposure to media news during the pandemic** (ref: Less than 30 min a day)		
Never	-0.227(0.472)	
Less than an hour a day	0.200(0.154)	
1–3 h a day	0.232(0.153)	
Four hours and more	0.460*(0.192)	
**Constants**	3.954*** (0.302)	3.103***(0.319)

Model 1 includes all variables in a single model, COVID-19 worry and sociodemographic, reported pre-pandemic stressors, habits of exposure to news, and COVID-19-related stressors. Model 2 includes socioeconomic, pre-pandemic stressors, and exposure to pre-pandemic news

[Ref] is Reference group; ^#^p < 0.1, * p < 0.05; ** p < 0.01; *** p < 0.001

#### Parents’ pre- COVID-19 worry

Model 2, Table 1 reveals that parents’ worry is modified by demographic characteristics, particularly gender. Lower worry levels were among male parents (p < 0.1) compared to female parents. Parents who never followed the news were less worried (p < 0.01). Increased worry was reported by parents with a history of worry symptoms (several days and every day of the week) (p < 0.05) and those with daily exposure to the news for 1- 3 h (p < 0.01).

#### Parents’ COVID-19-related worry by ethnic background

Table 2 depicts variables associated with parental worry adjusted by ethnic background. Worry among parents from “Asian and Middle-Eastern” backgrounds was positively associated with those who had a history of higher frequency of distress (p < 0.05) or worry (p < 0.1) symptoms and those with high health risk due to COVID-19 (p < 0.1). Whereas increased worry among Oceanian’s parents was associated with those perceiving lower neighbourhood safety (p < 0.05) and reporting excessive daily attention to pandemic news (one hour and over) (p < 0.05 and p < 0.01).

**Table 2:** Multiple regression analysis Variables associated with parents’ worry after controlling for ethnic background

Variable	Asian, Indian or Middle Eastern	Oceanian
***Sociodemographic:* Age (ref:** 25–35)		
35–44	-0.180 (0.222)	0.073 (0.224)
45–54	0.0238 (0.288)	0.304 (0.299)
55–65		0.669 (0.480)
**Gender (ref:** female)		
Male	0.224(0.246)	0.0749 (0.215)
**Education (ref:** High school or less)		
Vocational/technical	0.492 (0.740)	-0.418 (0.299)
Undergraduate	-0.059 (0.560)	-0.179 (0.240)
Graduate degree	0.0761 (0.567)	-0.049 (0.245)
**Household (ref:** $3,499 or less)		
3,500—$6,499	-0.115 (0.211)	0.146 (0.219)
$6,500 or more	-0.172 (0.247)	-0.200 (0.232)
**Life stressors (History): Safety perception (ref:** Very Safe)		
Quite Safe	-0.286 (0.210)	-0.409* (0.236)
Quite Unsafe	-0.310 (0.315)	-0.254 (0.228)
Not at all safe	0.030 (0.346)	-0.329 (0.371)
**History of parents’ distress (ref:** Not at all)		
More than half the day	0.713* (0.346)	-0.210 (0.320)
Several days	0.470* (0.231)	-0.241 (0.222)
Nearly every day	0.693 (0.497)	0.611 (0.380)
**History of parents’ worry (ref:** Not at all)		
More than half the day	-0.242 (0.497)	0.077 (0.416)
Several days	-0.413 (0.250)	0.425 (0.287)
Nearly every day	**0.813**^**#**^** (0.446)**	0.312 (0.562)
**COVID-19-related life stressors: Income change (ref:** No, it is about the same, and normally go to work)		
No, it is about the same, but work from home mainly	-0.417 (0.266)	0.040 (0.199)
Job-keeper	-0.549 (0.404)	-0.010 (0.346)
No Job	-0.146 (0.267)	0.346 (0.982)
Salary is reduced	-0.146 (0.267)	0.346 (0.982)
Seeking employment	-0.424 (0.377)	-0.136 (0.215)
**Exposure to COVID-19 infection** (**ref**: No direct exposure)		
Direct exposure (being infected or someone in the family being infected)	**-0.687**^**#**^** (0.360)**	0.178 (0.354)
“Having a close relationship with someone infected in the buildings or local area”	0.001 (0.370)	0.039 (0.335)
**Parents’ Exposure to Media news during the pandemic** (**ref**: Less than 30 min a day)		
Never	-0.383 (0.676)	-0.272 (0.597)
Less than an hour a day	0.126 (0.265)	0.305 (0.597)
1–3 h a day	0.168 (0.272)	0.663* (0.277)
Four hours and more	0.239 (0.311)	1.014** (0.354)
**Constants**	4.229*** (0.676)	2.839***(0.764)

[Ref] is Reference group; ^#^p < 0.1, * p < 0.05; ** p < 0.01; *** p < 0.001. Note:

The variance inflation factors (VIFs) in each independent variable was 2 or below, suggesting no redundancy or overlapping among variables (Chen et al., 2021). Figure S1, Additional File 1 visualises the pairwise potential correlation between study variables.

#### Variation over geography in variables contributes to parents’ worry

**Fig. 5:**
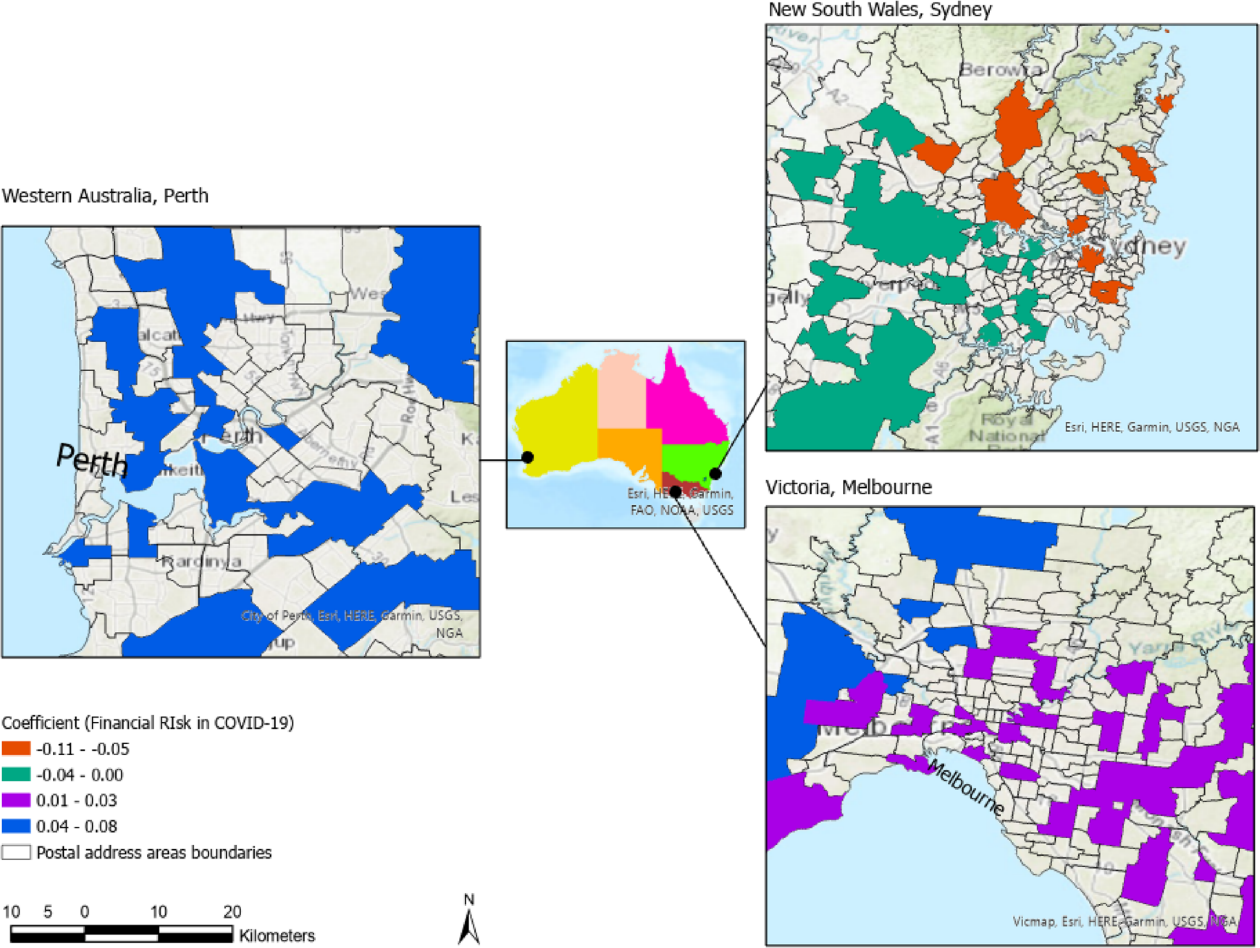
Spatial variation in the coefficient for income change contributing to parental worry in three states during COVID-19. The non-significant coefficient in WA denotes no effect of income change on parents’ worry while in NSW and VIC, financial risk has strongly contributed to varying parents’ worry

In a pre-to-during pandemic comparison, the spatial behaviour outcome of the GWR of parents’ worries for financial risk is shown in below Fig. 5. As recommended in public health studies, we categoirsed the coefficient into four quantiles [56]. Negative significance denotes that a decrease in explanatory variable level increases the impact on the response variable. The income changes during COVID-19 show coefficient values ranging from positive to negative (0.06-0.1). These coefficients displayed variation in the impact of the income change across states, revealing a significant positive effect in VIC but significantly negative effect in NSW and non-significant in WA.

### Variables’ importance

**Fig. 6:**
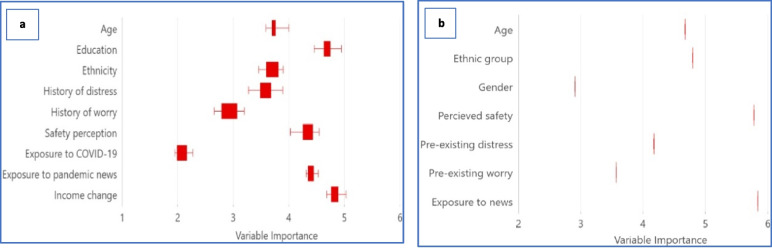
The outcome of the variables’ importance helps understand which variables drive parents’ worry. On the left, (**a**) The model used nine variables from previous global and local regressions to predict parents’ COVID-19 worry. On the right, (**b**) the model used seven variables found significant to predict parental pre-COVID-19 worries. Variable importance is determined using Gini coefficients

Figure 6 illustrates the variables’ importance in predicting parental worry using Forest-based classification and regression analysis. The input to the model were variables found significant in the multi-regression and the GWR.

In Fig. 6a, the most dominant variable in predicting parental COVID-19-related worry was the financial risk due to income changes. In parental pre-COVID-19 worry (Fig. 6b), the perception of neighbourhood safety shared equally the lead with media exposure in predicting parental worry. Additional details from the model can be found in Tables S4 and S5 in Additional File 1. The spatial geographical association between worried parents and children (Q4) shows a value of (0.21), indicating that children with heightened worry are primarily found within distraught parents (Table S6, Additional File 1). Examining the same relation before the pandemic yielded a value of 0.03 (closer to 0) indicating a lack of this association (Table S7, Additional File 1).

## Discussion

This study provides deeper insights into the variables that may contribute to varying parents’ perceived worry during the pandemic and how this varies across geography and time. Bivariate analysis of the varying level of worry before to during the pandemic reveals that the recent pandemic led to rising levels of parents’ worry and distress that may not have been restored to pre-pandemic. The study presents also several key findings.

First, *variables contributing to parents’ worry during abnormal events such as health crises vary from everyday life.* Most notably, parental worry during the pandemic varied by ethnic background, whereas this variable lacked predictive power in before the pandemic analysis. Worried Asian and Middle Eastern parents are explained by a history of worry or distress symptoms and increased health risk during the pandemic (direct exposure to the confirmed virus cases). This result is overwhelming yet aligns with existing knowledge that ethnic minority populations generally experience worse health outcomes than other groups during a pandemic [57]. A more prominent impact of the pandemic on black and brown communities is found in the US [14, 58, 59] and the UK [60, 61]. Health inequalities are likely due to a lower chance of primary health care and Medicaid access. However, McCaffery et al. [62] suggested that Australian people with inadequate health literacy and language barriers may experience more difficulty understanding government messages about the virus, making them less likely to adopt social distancing measures. The same study also suggests a higher endorsement of misinformation related to the pandemic among people whose English is not their first language.

Next, *exposure to stressors before COVID-19 may have helped parents develop coping strategies during the pandemic*. Our finding of links between worry and history of distress symptoms agrees with preceding research from various parts of the world. However, our analysis reveals that those who reported daily distress or worry before the outbreak were predictors of COVID-19 worried parents. Whereas, for pre-pandemic worry, mild to moderate symptoms of pre-existing distress were significant for parents who reported worry. Past studies agree that exposure to stressors over time may help individuals develop competencies and coping skills that promote resilience during crises such as this pandemic [19, 63].

Third, *while it is crucial to disseminate public health measures throughout the population, reliance on media news may (in)directly and inadvertently endanger public health in several ways.* Media news was critical in the recent pandemic in increasing people’s anxiety. Increased exposure to the news during times of uncertainty and crises is found in earlier research [64]. Media news playing a role in parents’ worries in our study agrees with previous studies on mass violence [17] and health crises like H1N1 [16]. It is also found in the recent pandemic in studies across the world [21, 34, 65].

Additionally*, using local regressions, we depicted the spatial variation in variables contributing to parental worry across time and geography.* The spatial behaviour of the income change variable is likely to mirror the varying policy and individual state actions. COVID-19 has evoked a massive global unemployment crisis. Despite Australia’s unemployment rate remaining among the lowest globally [66], it reached 7.5% in July 2020 (at the time of the survey), the highest since 1998. During the survey, about 1.6 million Australians were unemployed in July 2020. Approximately 6.7% (around 871,600 people) lost their jobs between March and May 2020 [67]. The highest employment decrease between March and October 2020 was in eastern states, with -4.1% in VIC and -1.3% in NSW. This likely explains the spatial behaviour and the significant coefficient for income change (Fig. 5) in eastern states. The lack of significance found in other states like WA (Fig. 5) may be due to the unemployment rate remaining unchanged in QLD, and being the lowest in WA and SA at -0.4% [67]. By May 2021, the government had reported $291 billion in support of businesses impacted by the pandemic. After announcing the state of emergency, the government introduced Job Keeper (Job Saver in NSW), a wage subsidy to allow employees to remain employed during the pandemic. However, the exclusion of some businesses from being qualifying for this scheme and the high mortality rates and the extended lockdown duration may have reduced the effect of these government support packages, particularly in VIC. These temporary schemes were cut in late September 2021, with reports indicating a drastic impact on the finance of Australian families’ households.

Also*, despite it bieng a health crisis, the amplified parental worry was triggered by factors other than a health risk.* Financial risk due to abrupt income changes during the pandemic was the leading variable contributing to parental worry (Fig. 6a). The perception of neighbourhood safety, a leading variable predicting parental worry before the pandemic, aligns with preceding studies linking neighbourhood-perceived safety with increased mental health concerns among adults [68] and parents [69].

Finally, *worried parents' geography aligns with the geographical patterns of children’s increased worry.* This finding aligns with past work suggesting a parental role in shaping children’s emotions [38, 70] and other analysis carried out using other method and conducted earlier by the author and published elsewhere [71].

There are some limitations of this study. Data collection occurred over two months but represented two points in time and making causal inferences difficult. Additionally, the study used online self-reported questionnaires with retrospective parent reports before COVID-19. The survey may be subject to selection bias, as participation requires respondents with internet access or who agreed to participate, making them less likely to report stress and psychological distress. To minimise the potential recall bias, the survey was administered a few weeks after the COVID-19 outbreak. However, some responses for pre-COVID-19 values were likely influenced by parents’ COVID-19 stress or other unmeasured biased, as we suspect in the case of the perceived safety variable (Fig. 5). This study also used items developed by the research team to assess factors influencing parental worry. At the time of the study onset, measures of COVID-19-specific stressors on parents and intervention strategies did not exist, although many are now available for future research. Despite every effort to represent the general Australian population, there is slight underrepresentation in some levels, such as parents’ education variable. Nevertheless, considering this study recruited parents who care for children in one age group (9–11 years) and compared with other studies on population worries in the US [19], this study’s sample size is well represented.

### Policy and research implications

The current findings provide important research-based evidence that can inform future preparedness for policy and guide further research direction. Uncertainty around health risks and economic concerns led research to anticipate that significant mental health needs would emerge in public during this pandemic [72]. However, this research shows that some families are more prone to experience worry than others. Public policies need to pay more attention to parents from minority ethnic backgrounds and those with a history of distress or worry symptoms. Providing awareness of the risks of excessive attention to pandemic news is also crucial. Parenting-specific strategies and in-state actions, mitigating financial and health risks, and providing community support to vulnerable parents may reduce unforeseen symptoms of parents’ depression and increase parents’ resilience. Increasing public health awareness to promote healthy ways of coping with increased worry and providing parents with specific information may improve families’ well-being. Given the potential for an impending mental health crisis, it is critical to ensure that parents have adequate access to mental health resources, including health services using the government health fund. Besides urging the government to take measures for short- and long-term mental health, it is important to ensure that the public is well informed about necessary information and minimises the spread of fake news.

The conceptual framework developed in this study can be used in similar research or other contexts, including the prevalence of worry due to natural disasters such as floods and bushfires. Future studies may examine the impact of variation in urban and rural settings on parents’ association with determinants of worry and address worry among those who care for older children. There is a dire need for a coordinated effort between researchers and policy-makers to further research in the spatial direction to generate parents’ spatial vulnerability index that can help prioritise resources to reduce COVID-19 impact on parents and elevate family well-being. Other research may examine the association between proximity to open green areas and parental worry.

## Conclusions

This study provides several novel insights. The study shed light on parents who care for primary school-aged children (9–11 years) and their vulnerability among those who experience worry as a window into family well-being. We conceptualised a framework to outline influencing factors and possible implications which may guide future research-based evidence in health crises or other contexts that adversely affects people’s well-being. The analysis reveals inequality in the impact of COVID-19 beyond physical health; variables like socioeconomics (ethnic background) have unevenly modified parental worry. The study depicted a variation in parental worry levels before-to-during the pandemic. Moreover, higher worry during health crises has been linked to factors other than physical health. Despite a very low percentage of the population being severely ill from COVID-19 rising parental worry reveals that living in repetitive stressful like extended enforced measures of lockdown and social distancing events has affected parents’ well-being. Yet, the effect vary in proximity to hotspot areas.

We found variations in the factors that most that are most important in predicting parents’ worry between everyday life and stressful health crisis events. In everyday life, parental worries are primarily predicted by neighbourhood safety, whereas during the pandemic, financial risk was the prime factor. Excessive exposure to news significantly contributes to parents’ worry at both times. Exposure to stressors before COVID-19 may have helped parents develop coping strategies during the pandemic. Finally the study depicted that worried children tend to be the spatially reside among worried parents.

We found variations in factors that amplified parents’ worry across geography. The variation in the variables contributing to parents’ worry across geography affirms that no one-size-fits-all model can identify vulnerable populations. Each country and state must identify target populations at greater risk and take adequate steps to mitigate the impact of worry across the family structure.

### Supplementary Information

**Additional file 1.**

## Data Availability

The data supporting the finding of this study are available from the corresponding author upon reasonable request.

## Abbreviations

WHO: World Health Organisation
COVID-19: Coronavirus disease 2019 SARS-COV-2
GIS: Geographic Information Systems
GWR: Geographic Weighted Regression
ABS: Australian Bureau Statistics
NSW: New South Wales
VIC: Victoria
SA: South Australia
WA: Western Australia
QLD: Queensland
US: United States
UK: United Kingdome
VIFs: Variance Inflation Factors
