# Supplementary material for: Insights into parents’ perceived worry before and during the COVID-19 pandemic in Australia: inequality and heterogeneity of influences
Source: BMC Public Health. 2023 Oct 7;23:1944. doi: 10.1186/s12889-023-16337-9 (PMC10559437; doi:10.1186/s12889-023-16337-9)
Supplement: Supplementary file 1 — Additional file 1. [file 12889_2023_16337_MOESM1_ESM.docx]

*Table S1. Detailed analytical techniques per research question*

| **Research questions** | **Methods** | | **Outcomes** |
| --- | --- | --- | --- |
| Descriptive analyses | Univariate and bivariate analysis | Scatterplot matrix, boxplot, bar charts | Understand sample data and pairwise variables relations |
| RQ1 | Model 1  Model 2  Model 3 | Global multilinear-regression model | - Variables associated with parents' worry about COVID-19 - Variables associated with parents' worry in pre-COVID-19 - Variables associated with parents' worry by ethnic background |
| RQ2 | GWR1 Pre-COVID-19  GWR2 In-COVID-19 | Local regression-Geographically Weighted Regression (GWR) | - Spatial variation of variables contributing to parents' pre-COVID-19 worry. - Spatial variation of variables contributing to parents' COVID-19-related worry |
| RQ3 | random forest algorithm models for pre-and during COVID-19 | Significant variables in Models 1 & 2 and GWR were input for | - Variables' importance |
| RQ4 | - Compared with earlier regression in children - Spatial association | | - Compare parents to children's most contributing predictor of worry - Spatial association of parents and children worries |

Table S2. Detailed subgrouping of countries that are under Oceanian ethnic background as per ABS

| **Broad group** | **Narrow group** | **Cultural and ethnic group** |  |  |
| --- | --- | --- | --- | --- |
| 1 OCEANIAN | 11 Australian Peoples | 1101 Australian |  |  |
|  |  | 1102 Australian Aboriginal |  |  |
|  |  | 1103 Australian South Sea Islander |  |  |
|  |  | 1104 Torres Strait Islander |  |  |
|  |  | 1105 Norfolk Islander |  |  |
|  |  |  |  |  |
|  | 12 New Zealand Peoples | 1201 Maori |  |  |
|  |  | 1202 New Zealander |  |  |
|  |  |  |  |  |
|  | 13 Melanesian and Papuan | 1301 New Caledonian |  |  |
|  |  | 1302 Ni-Vanuatu |  |  |
|  |  | 1303 Papua New Guinean |  |  |
|  |  | 1304 Solomon Islander |  |  |
|  |  | 1399 Melanesian and Papuan, nec |  |  |
|  |  |  |  |  |
|  | 14 Micronesian | 1401 I-Kiribati |  |  |
|  |  | 1402 Nauruan |  |  |
|  |  | 1499 Micronesian, nec |  |  |
|  |  |  |  |  |
|  | 15 Polynesian | 1501 Cook Islander |  |  |
|  |  | 1502 Fijian |  |  |
|  |  | 1503 Niuean |  |  |
|  |  | 1504 Samoan |  |  |
|  |  | 1505 Tongan |  |  |
|  |  | 1506 Hawaiian |  |  |
|  |  | 1507 Tahitian |  |  |
|  |  | 1508 Tokelauan |  |  |
|  |  | 1511 Tuvaluan |  |  |
|  |  | 1512 Pitcairn |  |  |
|  |  | 1599 Polynesian |  |  |
|  |  |  |  |  |
|  |  |  |  |  |

Source: Australian Bureau of Statistics, Australian Standard Classification of Cultural and Ethnic Groups (ASCCEG) 2019

*Table S3. Sample participants characteristics*

| **Domain** | **Variables** | **Levels** | n | % rounded |
| --- | --- | --- | --- | --- |
| Sociodemographic and economic background (Parents) | Gender | Male  Female | 148  191 | 43  57 |
|  | Ethnicity | Asian, Indian, and Middle Eastern  European  Oceanian  Others | 126  34  167  16 | 37  10  49  6 |
|  | Age | 25-34 years old  35-44 years old  45-54 years old  54-64 years old  undisclosed | 144  133  51  10  2 | 42.3  39.1  15  2.7  0.6 |
|  | Monthly household income | $3.499 or less  $3500 to $6,499  $6,500 or more  Undisclosed | 78  128  109  17 | 22.9  37.6  32  12 |
|  | Educational attainment | Vocational/Technical  High school or less  Undergraduate graduate (below bachelor's degree)  University graduate degree (at or above bachelor's degree)  Undisclosed | 28  52  129  131  39 | 8.2  15.2  37.9  38.5  11.5 |
|  | House with backyard | Yes  No | 295  44 | 86.7  12.9 |
| Demographic (Children) | Gender | Male  Female  Others  Undisclosed | 197  139  2  2 | 57.9  40.1  0.5  0.5 |
|  | Grades | Grade four  Grade five  Grade six | 127  124  89 | 37.3  36.4  26.1 |
|  | School type | Public  Private | 228  112 | 67  33 |


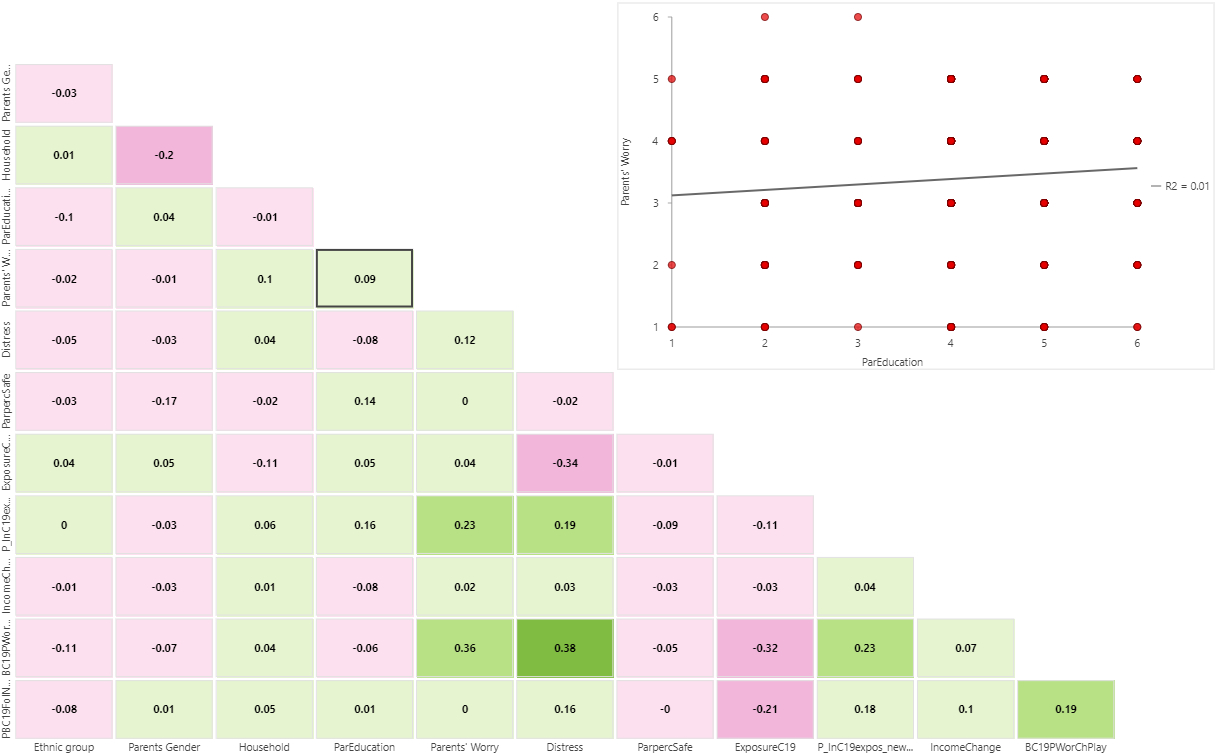


Figure S1. Scatterplot matrix grid showing the relationship in pairwise variables.

*Table S4. Forest-based classification model prediction of parents' worries using nine variables was found significant after adjusting for each state.*

**Model Characteristics**

| Number of Trees | 100 |
| --- | --- |
| Leaf Size | 1 |
| Tree Depth Range | 10-19 |
| Mean Tree Depth | 12 |
| % of Training Available per Tree | 100 |
| Number of Randomly Sampled Variables | 3 |
| % of Training Data Excluded for Validation | 10 |

**Top Variable Importance**

| **Variable** | **Importance** | **%** |
| --- | --- | --- |
| Parents income | 4.87 | 14 |
| Education | 4.75 | 14 |
| Exposure to news | 4.43 | 13 |
| Safety perception | 4.32 | 13 |
| Ethnic background | 3.78 | 11 |
| Parents age | 3.71 | 11 |
| History of distress | 3.42 | 10 |
| Parents worry before the pandemic | 2.79 | 8 |
| Health risk (exposure to cases) | 1.99 | 6 |

**Training Data: Classification Diagnostics**

| **Category** | **F1-Score** | **MCC** | **Sensitivity** | **Accuracy** |
| --- | --- | --- | --- | --- |
| A little Worried | 0.98 | 0.98 | 1.00 | 0.99 |
| Not at all worried | 0.95 | 0.95 | 0.91 | 0.99 |
| Quite worried | 0.98 | 0.97 | 0.98 | 0.99 |
| Very worried all the time | 0.99 | 0.99 | 0.98 | 1.00 |

*Predictions for the data used to train the model compared to the observed categories for those features

**Validation Data: Classification Diagnostics**

*Predictions for the test data (excluded from model training) compared to the observed values for those test features

Median Accuracy 0.333 was approximately reached at seed 852272

**Notes: The Out of Bag OOB errors** are calculated based on the percentage of incorrect classifications for each category among trees that did not see a subset of the trees in the forest. The percentage of incorrect OOB classifications for each category is printed in the geoprocessing messages. **The MSE** of the classifications is also printed and can be interpreted as the overall proportion of incorrect OOB classifications among all categories. **Sensitivity** for each category is reported as the percentage of times features with an observed category were correctly predicted. The **accuracy** diagnostic considers how well features with a particular category are predicted and how often other categories are miscategorised for the category of interest. It gives an idea about how frequently a category is identified correctly among the total number of confusions for that category. **Variable Importance** is calculated using **Gini coefficients** and can be thought of as the number of times a variable is responsible for a split and the impact of that split divided by the number of trees. Splits are each individual decision within a decision tree. **Variable importance** is a diagnostic that helps you understand which variables are driving the results of the model.

*Table S5. Model prediction using seven variables to predict parents' worry before the pandemic.*

##### Model Characteristics

| Number of Trees | 100 |
| --- | --- |
| Leaf Size | 1 |
| Tree Depth Range | 9-17 |
| Mean Tree Depth | 12 |
| % of Training Available per Tree | 100 |
| Number of Randomly Sampled Variables | 3 |
| % of Training Data Excluded for Validation | 10 |

##### Top Variable Importance

| **Variable** | **Importance** | **%** |
| --- | --- | --- |
| Safety | 4.86 | 14 |
| Exposure to news | 4.74 | 14 |
| Education | 4.55 | 13 |
| Ethnic background | 4.32 | 12 |
| Household income | 4.17 | 12 |
| History of distress | 3.69 | 11 |
| age | 3.61 | 10 |
| History of worry | 3.13 | 9 |
| Gender | 2.05 | 6 |

##### Training Data: Classification Diagnostics

| **Category** | **F1-Score** | **MCC** | **Sensitivity** | **Accuracy** |
| --- | --- | --- | --- | --- |
| A little Worried | 0.97 | 0.96 | 0.97 | 0.99 |
| Not at all worried | 0.98 | 0.97 | 1.00 | 0.99 |
| Not sure | 1.00 | 1.00 | 1.00 | 1.00 |
| Quite worried | 0.98 | 0.96 | 0.96 | 0.98 |
| Very worried all the time | 0.98 | 0.98 | 0.96 | 1.00 |

*Predictions for the data used to train the model compared to the observed categories for those features

*Table S6. Analysis details of spatial Association of Parents worry before and worry during the pandemic*

| Global Measure of Spatial Association  Global Correspondence of Input Zones within Overlay Zones  Global Correspondence of Overlay Zones within Input Zones  Number of Features from Input Zones  Number of Categories from Input Zones  Number of Features from Overlay Zones  Number of Categories from Overlay Zones | 0.2155  0.2633  0.1823  332  3  332  5 |
| --- | --- |

*Table S7. Analysis details of spatial association of children's worry of being sick and worry from playing out*

| Global Measure of Spatial Association  Global Correspondence of Input Zones within Overlay Zones  Global Correspondence of Overlay Zones within Input Zones  Number of Features from Input Zones  Number of Categories from Input Zones  Number of Features from Overlay Zones  Number of Categories from Overlay Zones | 0.0336  0.0334  0.0337  332  3  332  3 |
| --- | --- |
